# Application of Calcium Kinetics Characterization in Cardiac Disease Modeling and Drug Discovery

**DOI:** 10.3390/biom14070865

**Published:** 2024-07-19

**Authors:** Richard J. Roberts, Chi Keung Lam

**Affiliations:** Department of Biological Sciences, University of Delaware, Newark, DE 19713, USA

Calcium regulation is essential in virtually any cell due to its critical role as a second messenger in multiple signaling pathways. Numerous enzymes also rely on this cation for proper function [1]. In heart muscle, calcium is central in excitation–contraction (E-C) coupling, which dictates cardiac contraction and relaxation cycles [2,3]. Furthermore, calcium control in the mitochondria is crucial for cardiac energetics and cell death activation [4,5,6]. Thus, it is important to comprehensively understand how calcium can be regulated in cardiac cells and how specific calcium regulators can be employed to model cardiac diseases. This Special Issue “*Calcium Regulation in the Cardiac Cells*” promotes new studies to address this goal. 

**Figure 1:**
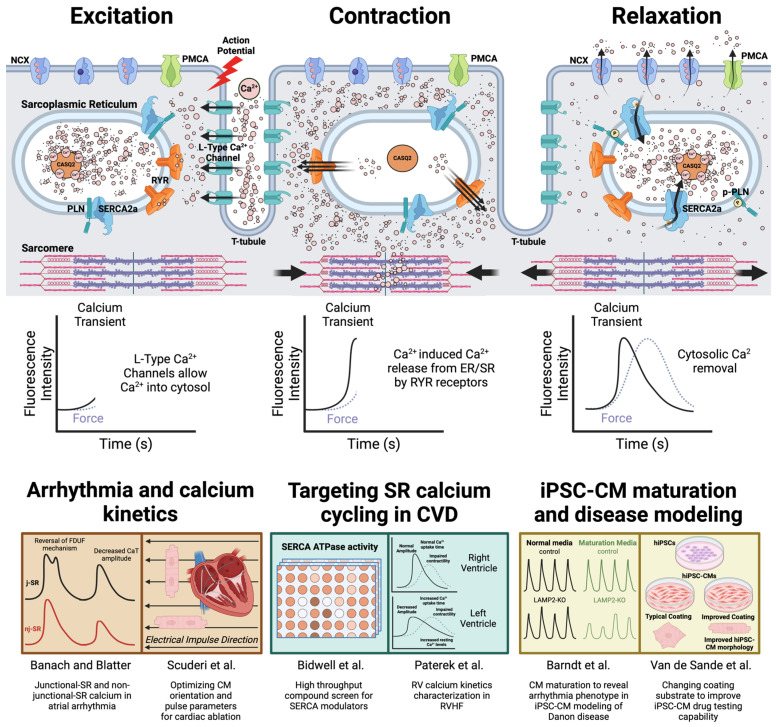
The application of characterizing cardiomyocyte excitation–contraction coupling in cardiac disease modeling and drug discovery. Membrane depolarization activates L-type calcium channel opening and allows calcium entry into the cardiomyocyte. These calcium ions bind to ryanodine receptors and activate SR calcium release (calcium-induced calcium release, or CICR), which then allows contraction. This calcium release in cardiomyocytes is easily detected by calcium imaging (indicated by the surge in calcium transient signal). Relaxation is enabled by removing cytosolic calcium via SR calcium ATPase (SERCA), plasma membrane sodium–calcium exchange (NCX), and plasma membrane calcium ATPase (PMCA). Calsequestrin (CASQ2) is the major calcium binding protein that absorbs calcium in the SR and increases SR calcium content. Removal of cytosolic calcium can be reflected by the decay phase in calcium imaging. The sequence of calcium cycling is maintained throughout an individual’s lifespan to ensure the continuous pumping action of the heart. Researchers can gather different mechanistic insights related to disease- or chamber-specific cell types by examining this sequence under different research conditions. Six manuscripts published in this Special Issue are summarized here [7,12,15,18,22,30].

All manuscripts in this Special Issue focus on the central role of calcium in E-C coupling in either disease modeling or target discovery. During E-C coupling, membrane depolarization triggers the opening of L-type calcium channels to allow calcium entry into the cytosol. These calcium ions then bind to ryanodine receptors and trigger their opening, causing more calcium release from the sarcoplasmic reticulum (SR). These rapid increases in calcium (reflected by the amplitude of each calcium transient in live cell imaging) facilitate crossbridge formation in the myofilaments for contraction. SR calcium ATPase (SERCA) and plasma membrane sodium–calcium exchanger (NCX) then remove cytosolic calcium (reflected by the calcium transient decay) to achieve muscle relaxation (Figure 1). This repetitive rise and fall of cytosolic calcium signals could be an excellent surrogate marker to reflect cardiomyocyte function. 

Paterek et al. examined differences in the calcium regulation of the left ventricle (LV) and right ventricle (RV) to understand the pathogenesis of RV dysfunction secondary to LV failure [7]. In this rat left ventricle myocardial infarction (MI) model, a marked decrease in RV function, subsequent to LV dysfunction, was observed in the hemodynamic study. However, unlike their LV counterparts, RV cardiomyocytes exhibit normal or even improved calcium cycling. Normally, failing cardiomyocytes exhibit reduced SERCA activity and rely more on NCX or plasma calcium ATPase to remove calcium for relaxation [8]. However, these features were not observed in the failing RV cardiomyocytes, suggesting that the RV dysfunction secondary to LV failure is not completely due to defects in E-C coupling. Furthermore, the notion that SERCA function is reduced in HF has recently been challenged [9]. This study provides additional insights into the need to identify the specific form of cardiac disease induced by SERCA dysfunction. 

Although the view that SERCA defect is a converging disease marker in all forms of HF is now being challenged, the benefit of activating or improving SERCA function in diseased hearts cannot be denied [10,11]. Thus, developing and identifying small molecules to enhance SERCA activity is an attractive approach to improve heart function. Bidwell et al. screened 46,000 compounds with an NADH-coupled high-throughput assay to identify hits that enhance SERCA calcium-dependent ATPase activity [12]. In a subsequent confirmation study using the gold standard oxalate-supported SR calcium uptake assay [13,14], 16 out of 19 identified compounds enhanced SR calcium uptake. This high-throughput platform is expected to facilitate research to improve SERCA characterization.

As the regular rise and fall of cardiomyocyte cytosolic calcium levels dictate the regularity of contraction and relaxation, calcium transients’ waveforms can also provide important information on the pathogenesis of arrhythmia. Banach and Blatter proposed the “Reverse fire-diffuse-uptake-fire (FDUF)” mechanism of SR calcium regulation as a contributor to atrial arrhythmia, such as atrial fibrillation (AF) [15]. With intrinsic differences in the lack of extensive transverse tubule (t-tubule) membrane system and ion channel expression, atrial cardiomyocytes are more prone to calcium alternans as they possess two different SR calcium stores: peripheral junctional (j-SR) and central non-junctional (nj-SR). Calcium release from j-SR plays a bigger role in triggering nj-SR calcium release, and calcium uptake from j-SR also contributes to calcium release from the nj-SR when the calcium signal propagates. This FDUF mechanism in atrial cardiomyocytes is prone to creating calcium alternans since disrupting this propagation sequence easily induces different calcium transient waveforms. In contrast, in ventricular cells, extensive t-tubule formation enhances simultaneous calcium release from various parts of the SR [16]. Furthermore, this type of calcium transient pattern can also be observed in cardiomyocytes derived from induced pluripotent stem cells (iPSCs) [17,18], which also lack extensive t-tubule structures [16,19]. Thus, establishing these FDUF and “reverse FDUF” mechanisms can have future applications outside of the atrial cardiomyocyte research.

Cardiac ablation is an AF treatment [20] and pulsed-field ablation is a developing method to serve this purpose [21]. Electroporation by high-voltage electric pulses transiently increases cardiomyocyte membrane permeability to hinder uncontrolled ion transport. Scuderi et al. attempted to identify the optimal combination of electrical strength, duration, and cell orientation for the best electroporation effect [22]. They developed a time-dependent nonlinear numerical model to simulate the effect of an electric field on the cardiomyocytes, which was then tested in vitro. They also employed calcium imaging to assess cell membrane permeability. Ultimately, they provided insights on how to optimize pulse field ablation in the future.

The ability of calcium imaging to reflect cardiomyocyte arrhythmia helps facilitate the application of iPSCs in cardiac disease modeling and drug discovery [23,24,25]. Since calcium kinetics and regulation in mature cardiomyocytes (isolated primary adult cardiomyocytes) differ from those of immature cells (such as iPSC-derived cardiomyocytes, or iPSC-CMs), characterizing the calcium transient waveform can provide tremendous information on the maturation state of experimentally used iPSC-CMs [26]. Barndt et al. took advantage of this property to assess whether metabolic maturation medium [27] can reflect hidden arrhythmic phenotypes in iPSC-CMs to model Danon disease [18]. While this disease is caused by mutations in lysosomal-associated membrane protein type 2 (LAMP2), an important regulator of lysosomal membrane in autophagy, patients also suffer from arrhythmia and sudden cardiac death [28,29], which warrants more research to dissect the underlying mechanisms. In this study, LAMP2-knockout iPSC-CMs did not exhibit arrhythmia in calcium imaging until the maturity of iPSC-CMs was improved. Their findings also suggested that calcium/calmodulin-dependent protein kinase IIδ over-activation may link LAMP2 defect to calcium dysregulation and arrhythmogenesis, providing a potential research direction for Danon disease treatment. As mentioned in the FDUF section, our understanding of these arrhythmic mechanisms may be even further improved by dissecting how j-SR and nj-SR calcium are regulated considering the lack of t-tubule structure in iPSC-CMs.

iPSC-CMs have been widely applied in assessing cardiotoxicity induced by small molecules [24]. Van de Sande et al. assessed whether enhancing structural maturity using a specific coating material, CELLvo™ Matrix Plus, can improve drug-induced cardiotoxicity in iPSC-CMs by examining the action potential, contractility, and transcriptomic changes [30]. While the maturation coating method enhanced morphology, altered action potential duration, and reduced contractility amplitude under basal conditions, it did not benefit the detection of drug-induced alteration in general. Since calcium imaging was not employed in their comprehensive assessment, it would be interesting to see if calcium kinetics can provide a different perspective to their conclusion. Furthermore, it would be very helpful to see more similar studies in the future to comprehensively compare various maturation methods [19] to offer more insights on improving the ability to detect and reflect physiological alteration using this iPSC platform.

This Special Issue presents six quality studies that demonstrate the crucial role of calcium regulation in studying disease mechanisms and exploring drug targets. It is exciting to see that these studies have laid a foundation to develop new directions for studying disease mechanisms, identifying optimal treatment conditions, or promoting new drug screening platforms. We look forward to seeing more studies that discover unknown regulators in cardiac calcium regulation and unravel new ways to analyze calcium signals in the future.
